# Neuroprotective Autophagic Flux Induced by Hyperbaric Oxygen Preconditioning is Mediated by Cystatin C

**DOI:** 10.1007/s12264-018-0313-8

**Published:** 2018-12-05

**Authors:** Zongping Fang, Yun Feng, Yuheng Li, Jiao Deng, Huang Nie, Qianzhi Yang, Shiquan Wang, Hailong Dong, Lize Xiong

**Affiliations:** 1 Department of Anesthesiology and Perioperative Medicine, Xijing Hospital, The Fourth Military Medical University, Xi’an, 710032 China; 2 Department of Gastroenterology, The First Affiliated Hospital of Xi’an Jiaotong University, Xi’an, 710061 China

**Keywords:** Stroke, Ischemia/reperfusion, HBO preconditioning, Cystatin C, Autophagy, Autophagic flux

## Abstract

We have previously reported that Cystatin C (CysC) is a pivotal mediator in the neuroprotection induced by hyperbaric oxygen (HBO) preconditioning; however, the underlying mechanism and how CysC changes after stroke are not clear. In the present study, we demonstrated that CysC expression was elevated as early as 3 h after reperfusion, and this was further enhanced by HBO preconditioning. Concurrently, LC3-II and Beclin-1, two positive-markers for autophagy induction, exhibited increases similar to CysC, while knockdown of CysC blocked these elevations. As a marker of autophagy inhibition, p62 was downregulated by HBO preconditioning and this was blocked by CysC knockdown. Besides, the beneficial effects of preserving lysosomal membrane integrity and enhancing autolysosome formation induced by HBO preconditioning were abolished in CysC^−/−^ rats. Furthermore, we demonstrated that exogenous CysC reduced the neurological deficits and infarct volume after brain ischemic injury, while 3-methyladenine partially reversed this neuroprotection. In the present study, we showed that CysC is biochemically and morphologically essential for promoting autophagic flux, and highlighted the translational potential of HBO preconditioning and CysC for stroke treatment.

**Electronic supplementary material:**

The online version of this article (10.1007/s12264-018-0313-8) contains supplementary material, which is available to authorized users.

## Introduction

Stroke is the second leading cause of mortality worldwide, causing 6.5 million deaths in 2013 [1]. It is also the major cause of lifelong disability, thereby a major concern in health care and neuroscience [2, 3]. Despite numerous efforts exploring drugs for ischemic stroke, no new neuroprotectant has been applied in clinical practice except for tissue plasminogen activator which was successfully introduced in 1995 [2–4].

Hyperbaric oxygen (HBO) preconditioning induces tolerance against ischemic neuronal injury, and is effective both *in vitro* and *in vivo* [5]. Owing to fewer clinical side-effects, HBO preconditioning has been under intense investigation [6]. Although neuroprotection is well defined, the mechanisms underlying HBO preconditioning remain elusive. Recently, we reported that Cystatin C (CysC), a lysosomal cysteine protease inhibitor, is a key mediator of the neuroprotection induced by HBO preconditioning [7]. However, how CysC changes after stroke and the mechanism underlying the neuroprotection mediated by CysC are unclear. In the current study, we investigated the changes of CysC after brain ischemia and explored the CysC-autophagy-neuroprotection linkage.

## Materials and Methods

All experiments were reviewed and approved by the Ethics Committee for Animal Experiments of the Fourth Military Medical University (Xi’an, China). Adult male Sprague-Dawley rats (8 weeks–10 weeks old, 250 g–300 g) were purchased from the University.

### Generation of CysC-knockout Rats

A genome editing technique based on transcriptional activator-like effector nucleases (TALEN) was used to produce CysC-knockout rats by targeting exon 1 of CysC [7]. To confirm the efficiency of CysC deletion, sequencing analysis was performed to detect base-pair deletions and western blot to assess the protein levels of CysC.

### HBO Preconditioning

Rats were randomly assigned to the control or HBO group. The rats in the HBO group were exposed to hyperbaric oxygen (2.5 atmospheres absolute and 100% O_2_, 1 h/day) for five consecutive days. The control rats were placed in the hyperbaric chamber with air at normal pressure.

### Transient Focal Cerebral Ischemia and Cerebral Blood Flow Estimation

Transient focal cerebral ischemia was achieved using a right middle cerebral artery occlusion (MCAO) model. Briefly, rats were anesthetized intraperitoneally (i.p.) with pentobarbital sodium (50 mg/kg). The right common carotid and external carotid arteries were exposed through a ventral midline neck incision. A monofilament (Beijing Sunbio Biotech Co., Ltd, Beijing, China) with a rounded tip was inserted into the common carotid immediately below the bifurcation. Then, it was advanced into the internal carotid ~ 18 mm–20 mm distal to the bifurcation until a mild resistance was felt, indicating occlusion of the middle cerebral artery. After 2 h, the middle cerebral artery was re-perfused by retracting the intraluminal monofilament. The regional cerebral blood flow of all rats subjected to ischemia/reperfusion (I/R) injury was monitored using a PF 5000 Laser Doppler Perfusion Monitoring Unit (PeriFlux 5000, Perimed AB, Stockholm, Sweden). The intervention was considered successful only if the regional cerebral blood flow sharply decreased to ≤ 30% of the baseline level after MCAO, and increased to ≥ 70% of the baseline level within 10 min of reperfusion.

### Intracerebroventricular Injection of CysC siRNA, Exogenous CysC and 3-Methyladenine

Twenty microliters of 20 μmol/L CysC siRNA or scrambled siRNA was infused into the right lateral ventricle 3 days before MCAO as reported previously [8]. The rats were anesthetized with pentobarbital sodium and placed in a stereotaxic apparatus. A burr hole was drilled into the skull 1.5 mm lateral and 1.0 mm posterior to bregma over the right hemisphere. A stainless-steel 26-gauge cannula (C315G, Plastic One, Roanoke, VA) was slowly introduced through the burr hole into the right lateral ventricle (3.8 mm beneath the dural surface). Reagents were infused into the ventricle at 0.5 μL/min. The CysC siRNA transfection complex was prepared according to the manufacturer’s instructions. The target sequence 5′-CCCAGACAAATTTGACTAACT -3′ was used.

3-Methyladenine (3-MA) (30 μg in 10 μL artificial cerebrospinal fluid, Sigma-Aldrich) was administered by intracerebroventricular injection 30 min before ischemia [8], while exogenous CysC (40 μg/kg) dissolved in artificial cerebrospinal fluid was delivered into the right lateral ventricle 30 min after reperfusion [7].

### Immunofluorescence Staining

Three hours post-reperfusion, the rats were deeply anesthetized before sequential perfusion with ice-cold saline and 4% paraformaldehyde in phosphate-buffered saline (PBS). The brains were removed and dehydrated in a sucrose gradient (20%–30%) in PBS at 4 °C. Coronal sections (10 μm thick) at bregma ± 2 mm were cut on a cryostat and stored at −20 °C until further use. The slices were blocked with 3% normal goat serum in 0.5% Triton X-100 for 30 min at room temperature. Subsequently, the slices were incubated with primary antibodies at 4 °C overnight, followed by incubation with secondary antibodies for 2 h at room temperature. The following antibodies were used: rabbit polyclonal anti-cathepsin B and mouse monoclonal anti-NeuN (both 1:1000, Abcam). DAPI (4’,6-diamidino-2-phenylindole; 1:1000; Sigma-Aldrich) was used to stain nuclei. The following secondary antibodies were used: green-fluorescent Alexa Fluor 488-conjugated donkey anti-mouse and red-fluorescent Alexa Fluor 594-conjugated donkey anti-rabbit (both 1:1000; Abcam). Fluorescent signals in the right penumbra region of cortex (2 mm lateral from the midline) were detected using confocal laser scanning microscopy (FV1000, Olympus, Tokyo, Japan).

### Western Blot

The designated region (penumbra: tissue less severely hypo-perfused than the ischemic core, and where neurons are functionally impaired but not yet irreversibly damaged) in the right cortex (between sections + 2 mm and − 3 mm of bregma) was separated after cold saline perfusion as described previously [9], and whole-cell protein was extracted using a total protein extraction kit (Merck Millipore). An equivalent of 40 µg protein from each sample was resolved on 12% SDS-PAGE. The following primary antibodies were used: anti-CysC (1:1000, Abcam), anti-LC3-I (1:1000, Sigma-Aldrich), anti-LC3-II (1:1000, Sigma-Aldrich), anti-Beclin 1 (1:1000, Cell Signaling), and anti-p62 (1:1000, Abcam). Goat anti-rabbit antibody was used as the secondary antibody (1:5000, Cell Signaling).

### Immuno-Electron Microscopy

Rats were anesthetized with 1% sodium pentobarbital (50 mg/kg i.p.) and perfused transcardially with 150 mL ice-cold 0.9% saline, followed by 500 mL cold mixture of 4% paraformaldehyde, 0.05% glutaraldehyde, and 15% (*v*/*v*) saturated picric acid in 0.1 mol/L phosphate buffer (pH 7.4) for 2 h. The brains were excised and fixed in the same fixative at 4 °C for 3 h. Serial coronal sections (50 μm) were cut on a vibratome (VT 1000S, Leica) and incubated overnight with the primary antibody rabbit anti-cathepsin B (1:100 in PBS containing 1% BSA). Subsequently, the sections were washed in PBS and incubated overnight with anti-rabbit IgG conjugated to 1.4 nm gold particles (Nanoprobes) at 1:100. After rinsing, the sections were post-fixed in 2% glutaraldehyde in PBS for 45 min. Silver enhancement was performed in the dark with an HQ Silver Kit (Nanoprobes) for visualization of cathepsin B immunoreactive signals. Before and after the silver enhancement step, the sections were rinsed several times with deionized water and incubated in ABC solution (Sigma-Aldrich) for 4 h, followed by visualization using the glucose oxidase-3,3′-diaminobenzidine method. The immunolabeled sections were fixed in 0.5% osmium tetroxide in 0.1 mol/L phosphate buffer for 1 h, dehydrated in an ethanol gradient and propylene oxide, and embedded in Epon 812 (SPI-CHEM, West Chester, PA). After polymerization, the sections were examined by light microscopy. Three sections containing cathepsin B immunoreactivity were selected from each rat, trimmed under a stereomicroscope, and mounted on new resin stubs. Ultrathin sections were cut on an ultra-microtome (EM UC6, Leica), mounted on mesh grids (6–8 sections/grid) followed by counterstaining with uranyl acetate and lead citrate, and observed in a JEM-1230 electron microscope (JEOL Ltd, Tokyo, Japan). Electron-micrographs were captured by a Gatan digital camera and analyzed with its software (832 SC1000, Gatan, Warrendale, PA).

### Statistical Analysis

SPSS 19.0 was used for statistical analysis. All data are presented as mean ± SEM and were analyzed using one-way ANOVA followed by the Dunnett or Bonferroni *post hoc* test. Differences were considered significant if the *P* value was < 0.05.

## Results

### HBO Preconditioning Enhances CysC Up-regulation After I/R Injury

**Fig. 1:**
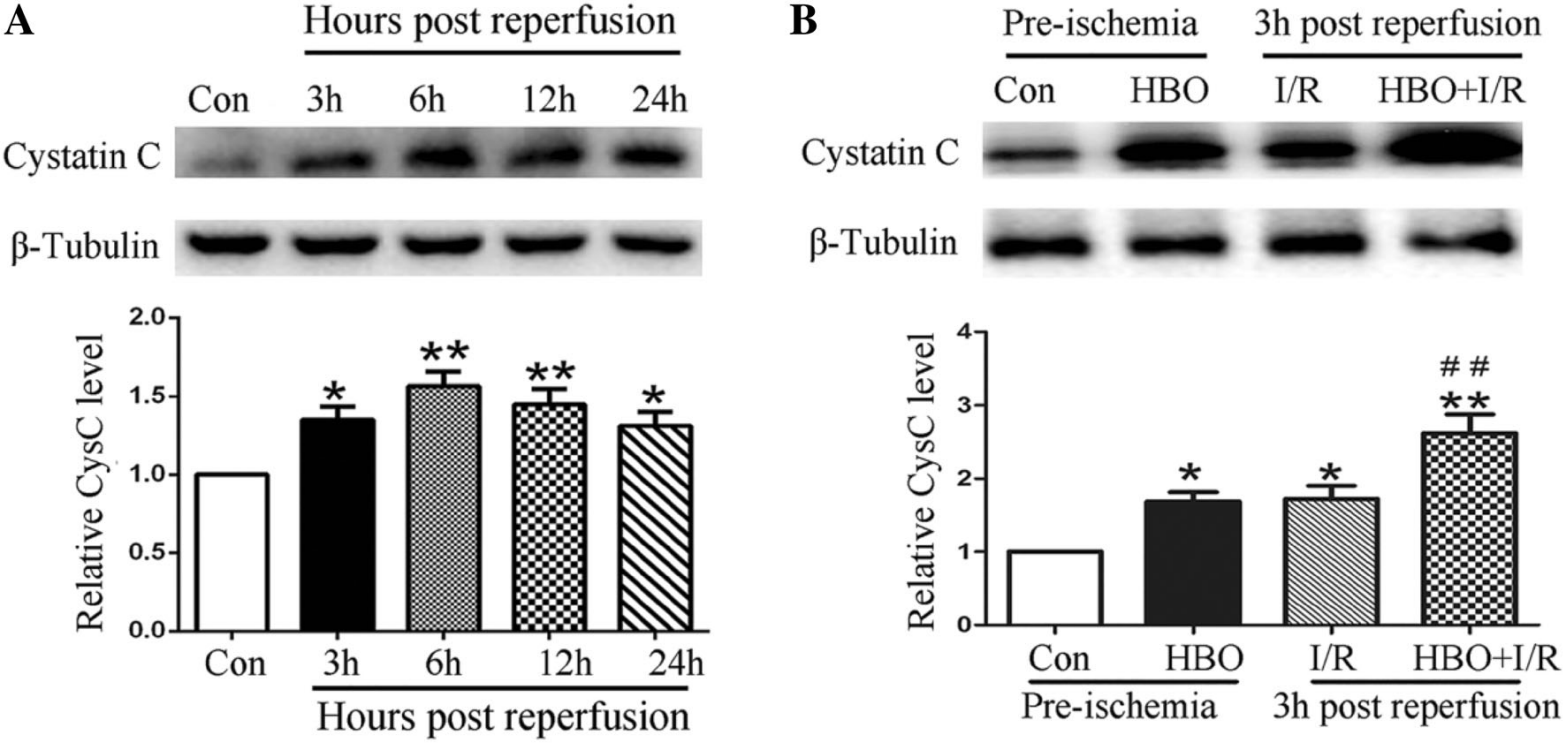
HBO preconditioning enhanced CysC up-regulation after I/R injury. **A** Representative immune-blots of CysC expression and statistics of Western blots showing that the CysC level was up-regulated after I/R injury (*P < 0.05, **P < 0.01 *vs* Control; one-way ANOVA followed by Dunnett’s test). **B** HBO preconditioning further enhanced the CysC increase at 3 h after I/R injury (*P < 0.05, **P < 0.01 *vs* Control; ^##^*P* < 0.05 *vs* I/R; one-way ANOVA followed by Bonferroni test; *n* = 6/group).

We have shown that the level of CysC is significantly increased after HBO preconditioning in non-ischemic brain [7]. To explore the role of CysC after ischemic injury, we first examined its expression at different time points after cerebral I/R. Compared to control rats without I/R injury, the expression of CysC in the ischemic penumbra was significantly up-regulated as early as 3 h after reperfusion, and the up-regulation was retained until 24 h post-reperfusion (Fig. 1A). Compared with the I/R group, HBO preconditioning further enhanced the expression of CysC at 3 h post-reperfusion [2.62 (0.26) *vs* 1.72 (0.18), *P* < 0.01, HBO + I/R *vs* I/R] (Fig. 1B). These results suggested that both ischemic injury and HBO preconditioning up-regulate CysC in the brain, while HBO preconditioning further enhanced this up-regulation induced by ischemia.

### Autophagic Flux Enhanced by HBO Preconditioning is CysC-Dependent

**Fig. 2:**
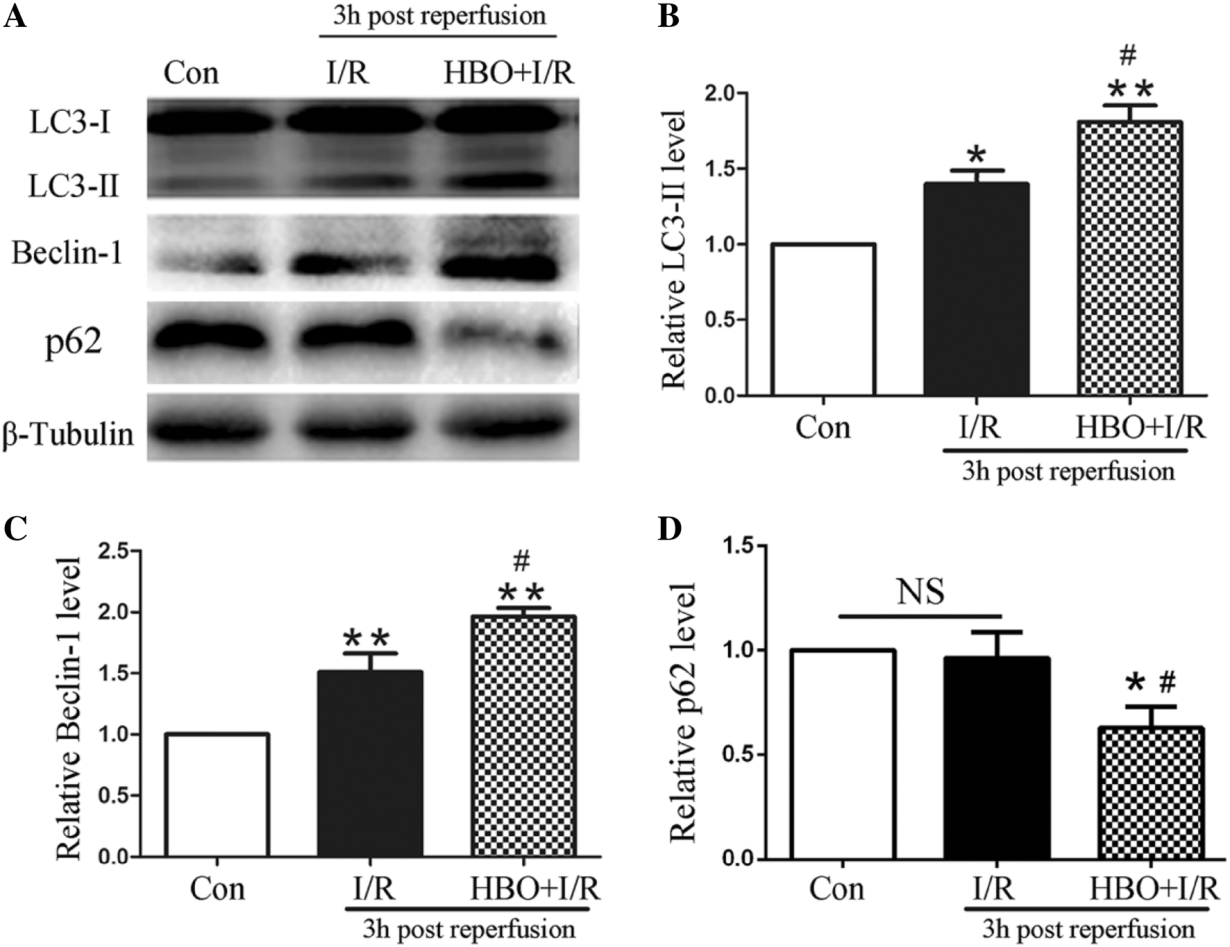
HBO preconditioning enhanced autophagic flux after I/R. **A** Representative Western blots of proteins associated with autophagic flux in rat brain. **B–D** Pooled data of LC3-II/β-Tubulin (**B**), Beclin-1/β-Tubulin (**C**), and p62/β-Tubulin (**D**) in the ischemic penumbra of rats at 3 h after reperfusion with/without HBO preconditioning (^*^P < 0.05, ^**^P < 0.01 *vs* Control, ^#^*P* < 0.05 *vs* I/R at 3 h post-reperfusion; one-way ANOVA followed by the Bonferroni test; *n* = 6/group).

Previous studies have demonstrated that both HBO preconditioning [8] and exogenous CysC promote the induction of autophagy [10]. So, we investigated the role of CysC in mediating autophagy induced by HBO preconditioning. First, we tested LC3-II and Beclin-1, which are reliable markers of autophagy induction. We found that both were significantly up-regulated 3 h after reperfusion [1.43 (0.12) normalized to control, *P *< 0.05; 1.51 (0.15), *P *< 0.01] (Fig. 2B, C). Interestingly, HBO-preconditioned rats exhibited a further elevation of these proteins [1.78 (0.13) for LC3-II and 1.96 (0.07) for Beclin-1 in the HBO + I/R group]. On the other hand, as a marker for autophagy inhibition, p62 was not changed after I/R; but HBO preconditioning significantly decreased the p62 level after I/R, indicating that autophagic flux was enhanced (Fig. 2D).

**Fig. 3:**
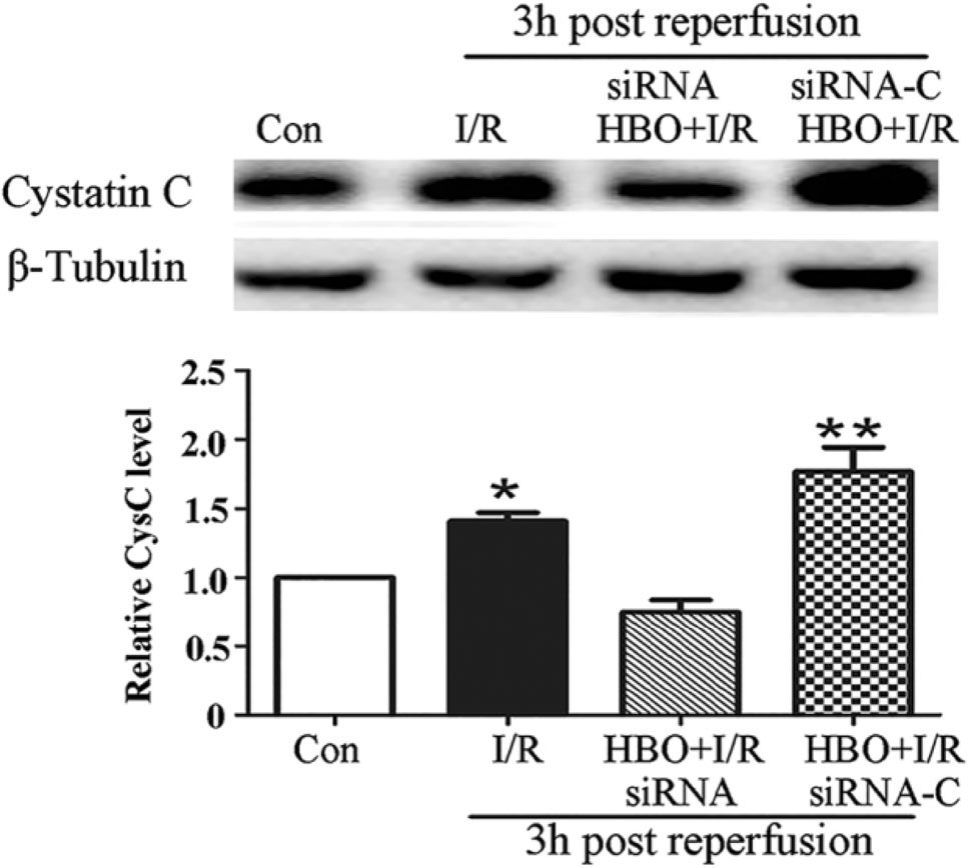
Short interfering RNA (siRNA) for CysC abolished the increase of CysC induced by ischemia and HBO preconditioning. Representative immunoblots (upper panel) and quantitative evaluation (lower panel) of CysC (*n *= 6/group). CysC expression was normalized to β-Tubulin expression (*P < 0.05, **P < 0.01 *vs* Control, one-way ANOVA with Dunnett’s correction; error bars represent SEM).

**Fig. 4:**
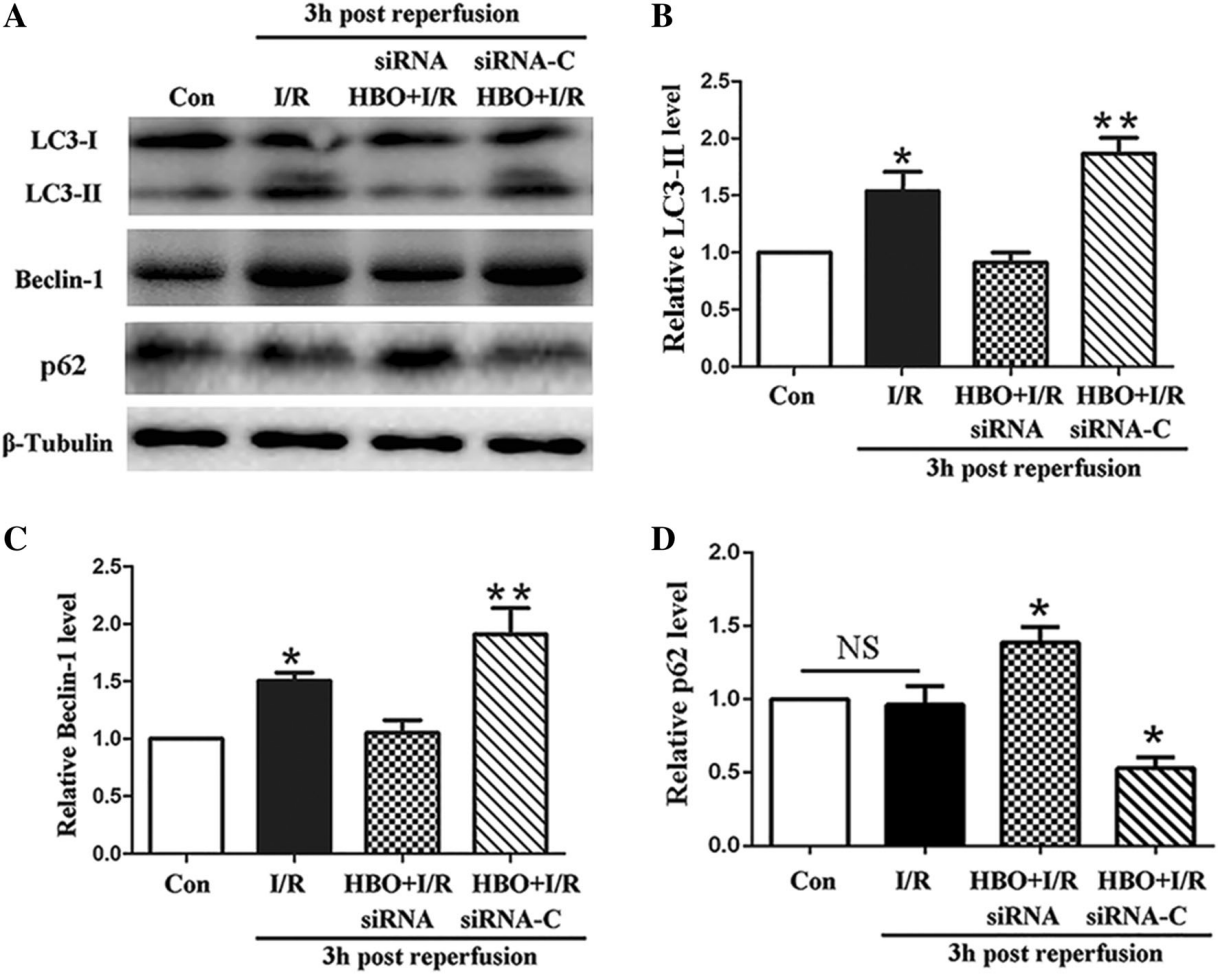
CysC is crucial in promoting autophagic flux induced by HBO preconditioning. **A** Representative Western blots of autophagy-related proteins in rat brain. **B–D** Pooled data of LC3-II (**B**), Beclin-1 (**C**), and p62 (**D**) in the ischemic penumbra of rats 3 h after reperfusion (*P < 0.05, **P < 0.01 *vs* Control; one-way ANOVA with Dunnett’s correction; *n* = 6/group).

To confirm the role of CysC, we knocked CysC down by intracerebroventricular administration of CysC siRNA. CysC expression was knocked down by 40% in rats without I/R and HBO preconditioning (Fig. S1). The increase of CysC induced by HBO preconditioning and I/R injury was significantly blocked by CysC siRNA (Fig. 3). Furthermore, knockdown of CysC significantly reduced the up-regulation of LC3-II and Beclin-1 induced by HBO preconditioning and I/R injury (Fig. 4A–C), while the decrease in p62 induced by HBO preconditioning and I/R injury was reversed (Fig. 4D).

**Fig. 5:**
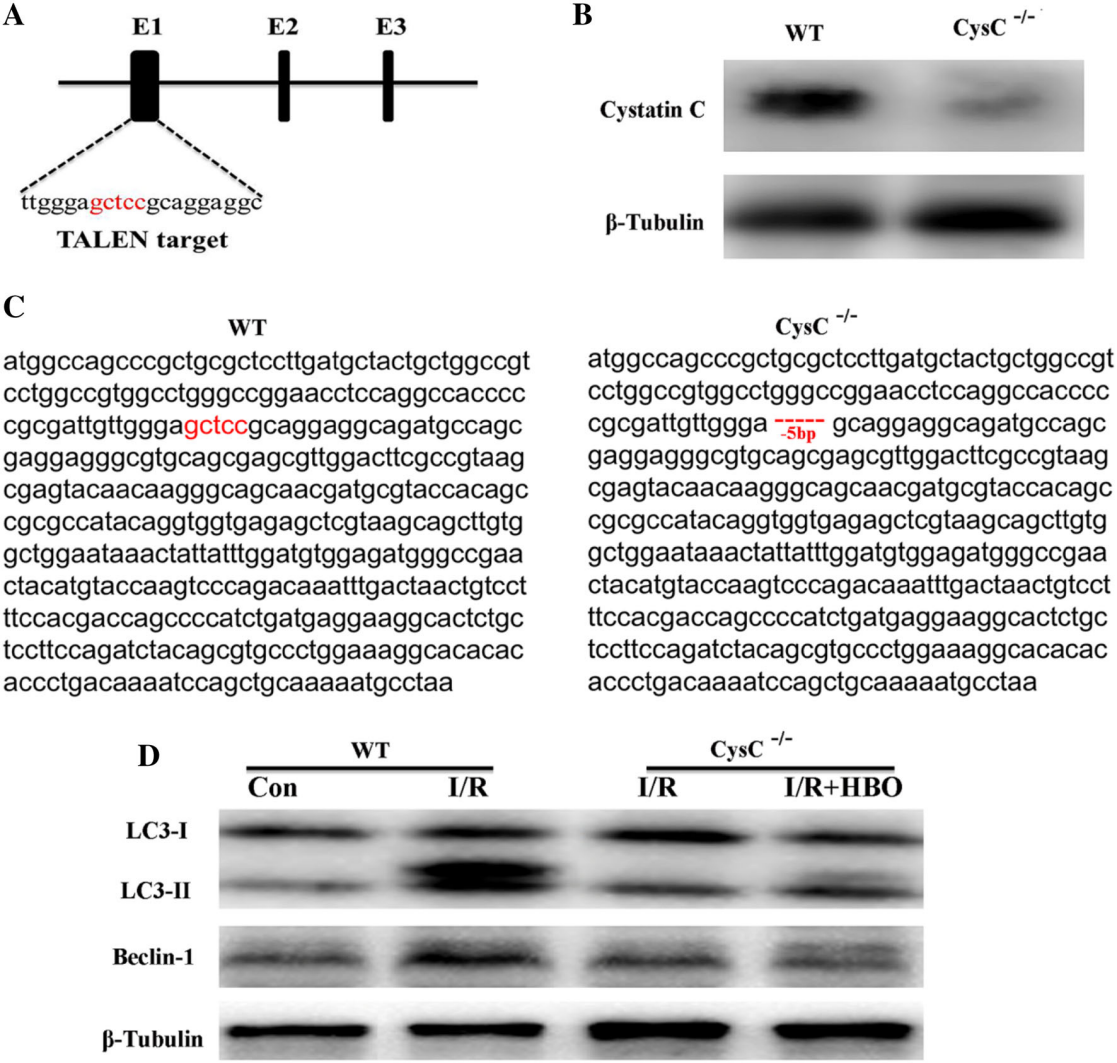
Up-regulation of LC3-II and Beclin-1 was abolished in CysC^−/−^ rats. **A** Schematic overview of the TALEN-based genome-editing strategy used to generate CysC-knockout rats. **B** Representative Western blots of CysC from rat brain. **C** Representative coding sequences of wild-type and mutated alleles identified from clonal amplicons. Red dashes indicate the deleted bases. **D** Representative Western blots of LC3-II and Beclin-1 from the ischemic penumbra of rats 3 h after reperfusion.

To corroborate the role of CysC, we generated CysC^−/−^ rats using TALEN-based genome-editing to target exon 1 of CysC (Fig. 5A). Sequencing analysis of the targeted CysC locus revealed a 5-bp deletion of the coding sequence (Fig. 5C). Western blot analysis confirmed the gene-targeting efficiency of CysC, demonstrating a significant reduction in CysC expression (Fig. 5B). Compared to the non-ischemic control group, I/R injury increased LC3-II and Beclin-1 in wild-type rats. However, in CysC^−/−^ rats, the up-regulation of LC3-II and Beclin-1 induced by HBO preconditioning and I/R injury disappeared (Fig. 5D). These results indicated that CysC is an essential mediator in promoting the autophagic flux induced by HBO preconditioning and cerebral ischemia.

### CysC Preserved Lysosomal Membrane Integrity and Prompted Autolysosome Formation

**Fig. 6:**
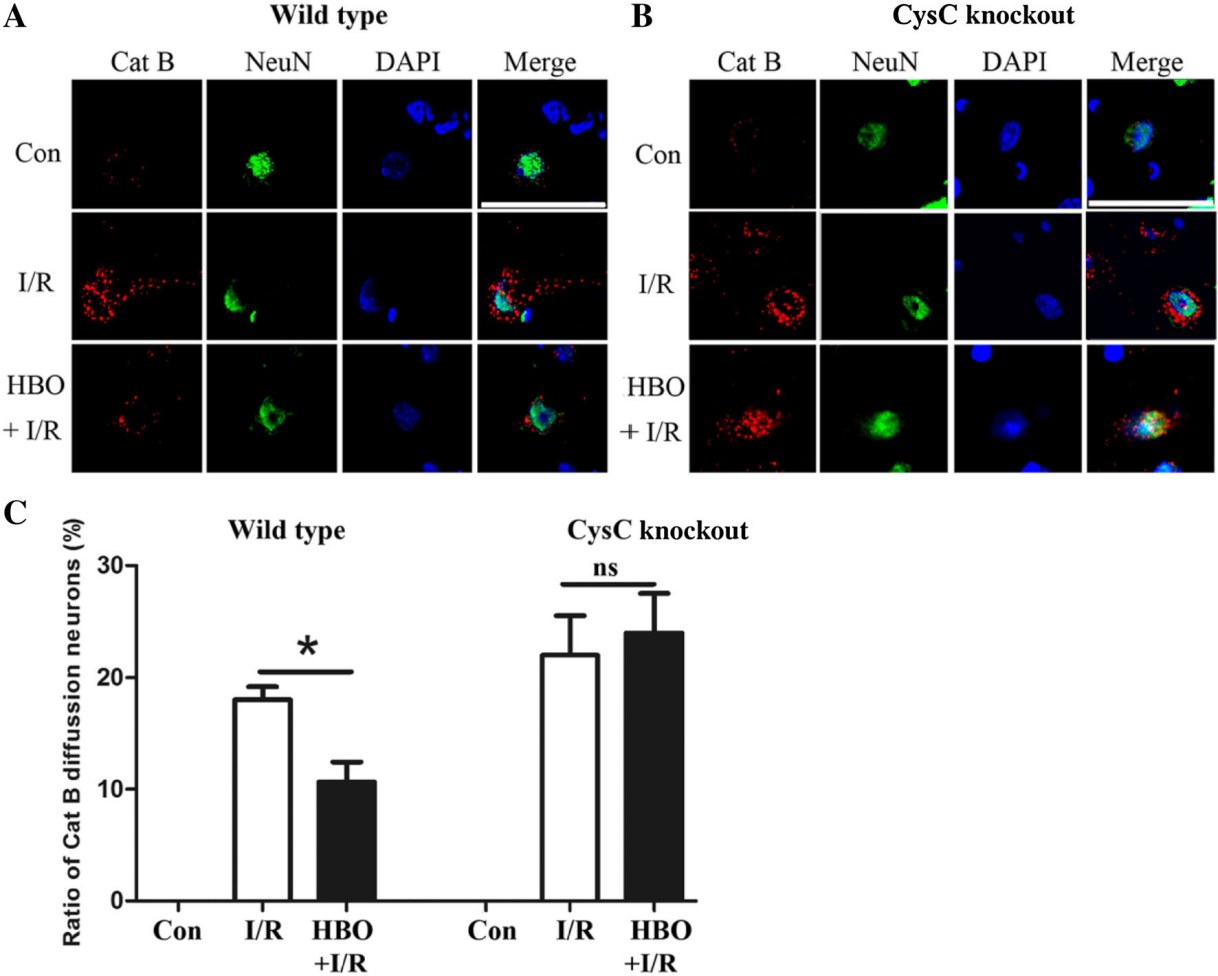
CysC is critical in preserving the lysosomal membrane integrity induced by HBO preconditioning. **A** Immunofluorescence of the subcellular localization of cathepsin B (red) with the neuronal nuclear marker NeuN (green) in the ischemic penumbra at 3 h post-reperfusion. The cytoplasmic and nuclear diffusion of cathepsin-B immunoreactivity indicated lysosomal leakage in the rats receiving MCAO. HBO preconditioning reduced this leakage of cathepsin B. **B** In CysC^−/−^ rats, the beneficial effect of reducing cytoplasmic cathepsin-B diffusion induced by HBO preconditioning was reversed. Scale bars, 50 μm. **C** Quantitative analysis of neurons with diffuse cathepsin B. Twenty-four cells per rat were examined, and 3 rats were used in each group.

Autophagic processing, also described as autophagic flux, includes sequestration of the cytoplasmic cargo into double-membrane vesicles and delivery of the autophagosome to the lysosome for degradation, emphasizing the crucial role of the lysosome during autophagy [11]. As shown in Fig. 6A, in sham-operated wild-type (WT) rats, cathepsin B was observed as fine, granular, peri-nuclear immunoreactivity, indicating intact lysosomal membrane integrity. Three hours post-reperfusion, cathepsin B increased and was diffusely distributed throughout the cytoplasm and nucleus, whereas HBO preconditioning alleviated the diffusion. Meanwhile, we found that in CysC^−/−^ rats, the improvement of lysosomal membrane integrity induced by HBO preconditioning was totally reversed (Fig. 6B, C). Taken together, these results suggested that the disruption of lysosomal integrity after I/R injury is alleviated by HBO preconditioning in a CysC-dependent manner.

**Fig. 7:**
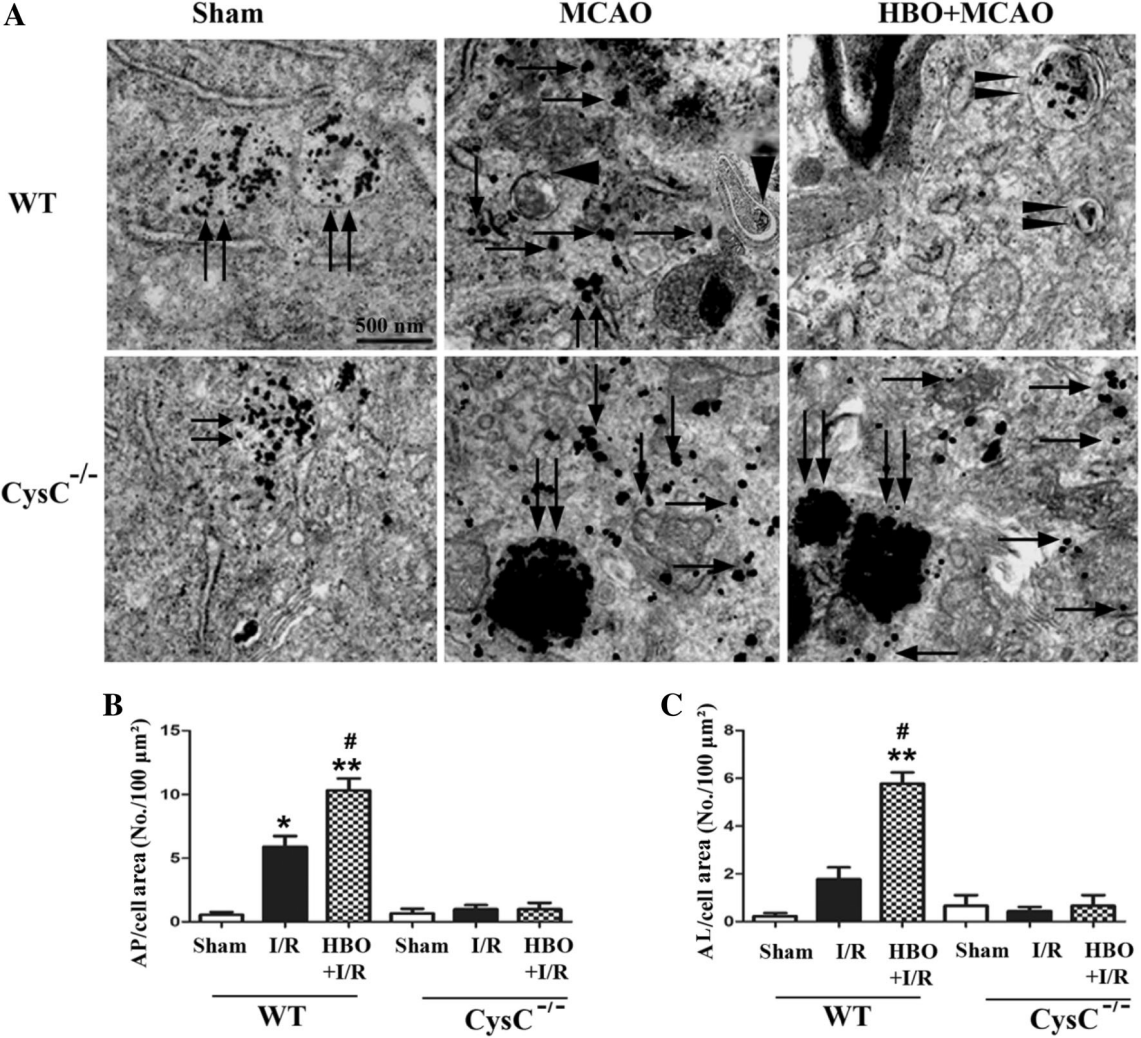
Knockout of CysC reversed the preservation of lysosomes and formation of autolysosomes induced by HBO preconditioning. **A** Representative immuno-electron micrographs of neurons 3 h after I/R, indicating increased numbers of autolysosomes in the HBO preconditioning group of WT rats, but this was reversed in CysC knockout rats. Double arrows, lysosomes with clusters of immuno-gold particles within a single membrane structure; arrowheads, autophagosomes with a double membrane structure comprising phagocytic material; double arrowheads, autolysosomes showing lysosomes encompassing the phagocytic membrane structure; arrows, cytoplasmic diffusion of cathepsin B from lysosomes, representing disruption of lysosome integrity. Scale bar, 0.5 μm. **B, C** Quantitative analysis of autophagosomes (AP, **B**) and autolysosomes (AL, **C**) (*P < 0.05, **P < 0.01 *vs* WT Sham; ^#^*P *< 0.05 *vs* WT I/R; 9 cells from 3 rats in each group were examined).

To visualize autophagic flux directly, cortical neuronal ultrastructure was observed with the aid of immuno-electron microscopy. As shown in Fig. 7A, in sham-operated WT rats, cathepsin B was wrapped within the lysosome, where immunogold particles clustered within a single membrane structure. Three hours after reperfusion in the WT rats without HBO preconditioning, a number of autophagosomes could be seen in the neurons of the penumbra, while autolysosomes were rarely detected. Moreover, the immunogold silver-enhanced cathepsin B staining was diffusely located in the cytosol, indicating the disruption of lysosomes. HBO preconditioning significantly attenuated the damage of lysosomes caused by ischemic injury in WT rats, as revealed by fewer immunogold particles outside the lysosome. Interestingly, autolysosomes (lysosomes containing a phagocytic membrane structure cargo) were evident in the HBO preconditioning group. In contrast, HBO preconditioning neither rescued the disruption of lysosomes nor promoted the formation of autolysosomes in CysC^−/−^ rats (Fig. 7B, C). These results indicated that CysC is essential in preserving lysosomal integrity and forming autolysosomes, thereby promoting the completion of autophagy.

### 3-Methyladenine Attenuated the Neuroprotective Effect of Exogenous Cystatin C against Focal Cerebral Ischemic Injury

**Fig. 8:**
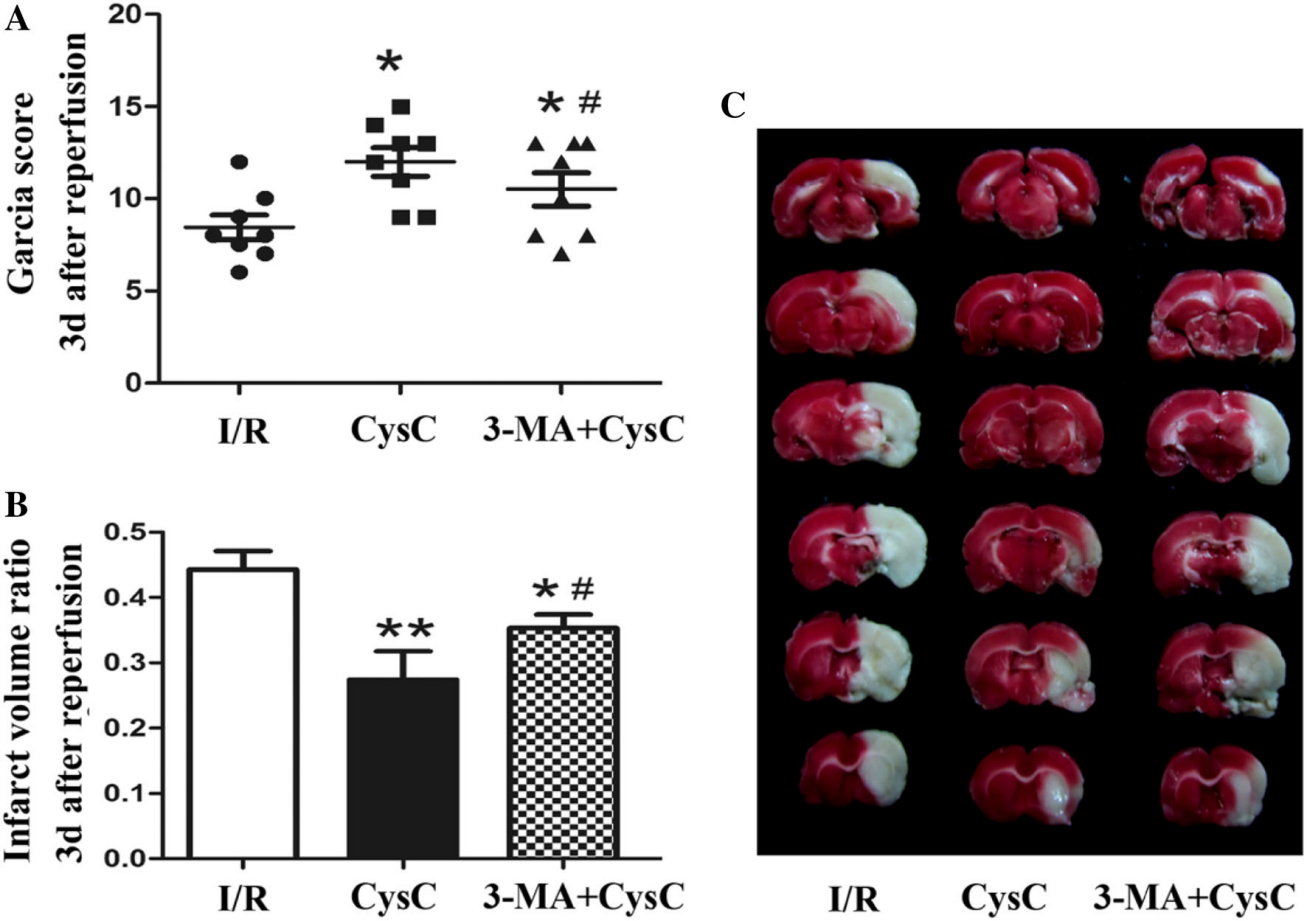
**A** Neurobehavioral score 72 h after reperfusion (*n* = 8/group). CysC improved the neurobehavioral score, and 3-methyladenine (3-MA) partially reversed the beneficial effect of CysC. **B, C** Representative 2,3,5-triphenyltetrazolium chloride-stained brain sections (**C**) and quantitative evaluation (**B**) of the infarct volume 72 h after reperfusion. CysC reduced the infarct volume, while 3-MA attenuated the protective effect of CysC (*P < 0.05, **P < 0.01 *vs* I/R; ^#^*P *< 0.05 *vs* CysC). I/R, animals subjected to MCAO for 120 min; CysC, animals received 40 μg/kg exogenous CysC 30 min after reperfusion; 3-MA was delivered by intracerebroventricular injection 30 min before ischemia.

The above results indicated that the elevation of CysC induced by HBO preconditioning enhances autophagy. However, direct evidence linking CysC-autophagy-protection was still missing. Therefore, we used 3-MA to determine whether CysC can still confer the neuroprotection in the absence of autophagy. Exogenous CysC significantly improved neurological function and reduced the infarct volume 72 h after reperfusion compared with the I/R group receiving saline (Fig. 8). The neurobehavioral score of the 3-MA + CysC group was higher than that of the I/R group and lower than that of the CysC group (*P* < 0.05). The infarct volume in the 3-MA + CysC group was smaller than that in the I/R group and larger than that in the CysC group. These results revealed that the autophagy inhibitor partially reversed the neuroprotection induced by CysC.

## Discussion

Our results, for the first time, showed that the expression of CysC was elevated in the penumbra after ischemic injury and HBO preconditioning further enhanced this elevation. Since CysC started to increase as early as 3 h after I/R and our previous research had demonstrated that the elevation of LC3-II and Beclin-1 also occurred as early as 3 h [8], we selected 3 h as the time point. LC3-II and Beclin-1 exhibited an increase similar to CysC. Conversely, HBO preconditioning significantly decreased the p62 protein level. Moreover, knocking down CysC decreased the upregulation of LC3-II and Beclin-1, while reversing the downregulation of p62. Furthermore, HBO preconditioning preserved the integrity of the lysosome membrane and enhanced the formation of autolysosomes in WT rats. These advantages of HBO preconditioning disappeared in CysC^−/−^ rats. Above all, our results provided both biochemical and morphological evidence that CysC induced neuroprotection against ischemic injury through enhancing autophagic flux.

CysC is a potent endogenous inhibitor of lysosomal cysteine proteinases. Notably, it is abundant in the central nervous system, emphasizing its crucial role in the brain [12]. A recent study demonstrated that CysC prevents oxidative injury [10]. Exogenous CysC is neuroprotective by reducing the infarct volume in ischemic stroke in rats [13]; this phenomenon is in agreement with our recent result [7]. However, besides the maintenance of lysosomal integrity [7], little is known about the mechanism of elevated CysC in mediating the neuroprotection induced by HBO preconditioning.

As a lysosomal cysteine protease inhibitor, CysC is crucial for lysosomal function and is closely associated with autophagy [10, 12, 14], which is a vital self-repair process for neuronal survival after ischemic injury [15]. Furthermore, autophagy involves the processes of induction, delivery of autophagy substrates to lysosomes, and degradation of substrates inside lysosomes, defined as “autophagic flux” [16]. Previously, we demonstrated that HBO preconditioning enhances the induction of autophagy, such as up-regulating LC3-II/LC3-I and Beclin-1 [8]. Recently, we demonstrated that CysC is critical in maintaining lysosomal integrity [7]. However, it had neither been determined whether HBO preconditioning affects autophagic flux after stroke nor the function of CysC in mediating autophagy induced by HBO preconditioning. Here, we evaluated autophagic flux by studying the levels of LC3-II, Beclin-1, and p62, which are essential markers for the induction and degradation of autophagy, respectively. We demonstrated that HBO preconditioning not only promoted the induction, but also enhanced the degradation in a CysC-dependent manner. It should be noted that our results demonstrated that CysC was essential for the upregulation of Beclin-1, which is the mammalian orthologue of yeast Atg6 and plays a central role in autophagy [17]. As a component of the vps34 complex, Beclin-1 interacts with several cofactors (Atg14L, UVRAG, Bif-1, Rubicon, Ambra1, IP3R, PINK, and survivin), thereby regulating many major steps in autophagic pathways, from autophagosome formation to autophagosome/endosome maturation [17]. Besides, as a novel Bcl-2-homology (BH)-3-only protein, Beclin-1 interacts with members of the anti-apoptotic Bcl-2 family [18]. Therefore, CysC may play multiple roles in enhancing autophagic flux and regulating apoptosis. It is important that future studies assess the precise roles of CysC and Beclin-1 in autophagy and apoptosis.

Tizon *et al*. reported that CysC modulates autophagy induction through mTOR under multiple neuronal challenges *in vitro* [10], which is in line with our current findings *in vivo*. On the other hand, the process of autophagy requires functional lysosomes to degrade the autophagosomal cargo [19]. In the current study, CysC^−/−^ rats were used to show that CysC was pivotal in preserving lysosomal membrane integrity against brain ischemia. Furthermore, our immuno-electron microscopic results in WT and CysC^−/−^ rats indicated that CysC was essential in promoting the formation of autolysosomes, which is the key feature of a completed autophagic process. However, although our results indicated that CysC affects the protein levels of LC3-II, Beclin-1, and p62, the underlying mechanism remains to be clarified. Besides, the roles of CysC in other autophagy-regulating molecules, such as mTOR and ULK1/2, need further exploration.

To confirm the role of autophagy in the neuroprotection that results from CysC, an intracerebroventricular injection of 3-MA, which suppresses the formation of autophagosomes by inhibiting class III phosphatidylinositol 3-kinase [8], was administered 30 min before ischemia. The results showed that exogenous CysC induced robust neuroprotection against ischemic injury, which is in accord with our previous results [7]. Besides, 3-MA attenuated the neuroprotective effect of exogenous CysC. This result is in agreement with a previously published study showing that 3-MA inhibits the protective effect of CysC by blocking the activation of autophagy *in vitro* [10]. However, as 3-MA only compromised, but did not block the neuroprotection induced by CysC, it is likely that CysC has multiple neuroprotective actions other than enhancing autophagy.

Nevertheless, the current study has some limitations. First, we did not explore the effect of exogenous CysC in promoting autophagy during cerebral ischemia. Second, the mechanism underlying the CysC-enhanced autophagic flux, including autophagy induction, degradation, and lysosome preservation is not clear. In our latest study we found that transcription factor EB, a master regulator of lysosomal biogenesis and autophagy, was dramatically lower in CysC^−/−^ rats (data not shown). This phenomenon may to some extent explain why CysC affected the entire process of autophagic flux, but this need further exploration.

In summary, using gene manipulation technology, we demonstrated that CysC is a determinant for the neuroprotective effect of HBO preconditioning through promoting autophagic flux in the brain after ischemia. Our findings further clarify the mechanism underlying the neuroprotection of HBO preconditioning and CysC; this may facilitate their clinical application.

## Electronic supplementary material

Below is the link to the electronic supplementary material.

Supplementary material 1 (PDF 605 kb)
