# Supplementary material for: Neuroprotective Autophagic Flux Induced by Hyperbaric Oxygen Preconditioning is Mediated by Cystatin C
Source: Neurosci Bull. 2018 Dec 5;35(2):336–46. doi: 10.1007/s12264-018-0313-8 (PMC6426805; doi:10.1007/s12264-018-0313-8)
Supplement: Supplementary file 1 — Supplementary material 1 (PDF 605 kb) [file 12264_2018_313_MOESM1_ESM.pdf]

Supplementary Material

Neuroprotective Autophagic Flux Induced by Hyperbaric Oxygen Preconditioning is Mediated by Cystatin C

Zongping Fang[1,#], Yun Feng[2,#], Yuheng Li[1,#], Jiao Deng[1], Huang Nie[1], Qianzhi Yang[1], Shiquan Wang[1], Hailong Dong[1,*], Lize Xiong[1,*]

1. Department of Anesthesiology and Perioperative Medicine, Xijing Hospital, The Fourth Military Medical University, Xi'an 710032, China

2. Department of Gastroenterology, The First Affiliated Hospital of Xi'an Jiaotong University, Xi'an 710061, China

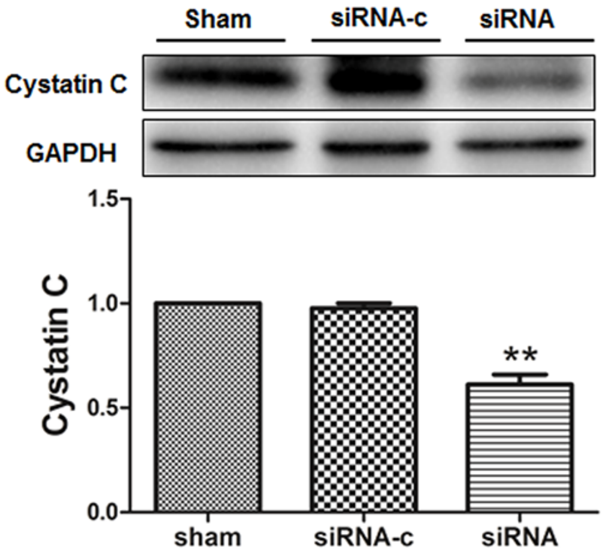

Fig. S1. Efficiency of intracerebroventricular CysC siRNA injection on CysC expression in the brain (n = 4/group). **$P < 0.01$ *vs* sham.
